# Comparative study of anterior segment measurements using 3 different instruments in myopic patients after ICL implantation

**DOI:** 10.1186/s12886-019-1194-y

**Published:** 2019-08-13

**Authors:** Ting Wan, Houfa Yin, Yi Yang, Fang Wu, Zhiyi Wu, Yabo Yang

**Affiliations:** Eye Center, Second Affiliated Hospital, School of Medicine, Zhejiang University, 88 Jiefang Road, Hangzhou, 310009 China

**Keywords:** Implantable Collamer lens, Anterior chamber depth, Vault, Optical coherence tomography, Ultrasound biomicroscopy, Pentacam

## Abstract

**Background:**

To compare and correlate anterior segment measurements of myopic eyes implanted with Implantable Collamer lens (ICL V4c) by using anterior segment optical coherence tomography (AS-OCT), Pentacam and ultrasound biomicroscopy (UBM).

**Methods:**

Anterior chamber depth (ACD), distance between corneal endothelium and anterior surface of ICL(C-ICL) and central vault were measured in 82 phakic myopic eyes of 82 patients who underwent ICL surgery, by using AS-OCT, Pentacam and UBM consecutively at 3 months follow up. The correlation and agreement of instruments were accessed by using Intraclass correlation coefficient (ICC) and the Bland-Altman plot.

**Results:**

AS-OCT showed higher ACD, C -ICL and central vault measurements than both of Pentacam and UBM (*P* < 0.001), while Pentacam showed lower measurements than UBM (*P* < 0.05). The Pearson correlation coefficient (r) was 0.91 to 0.96, and ICC was 0.95 to 0.98 for all measurements between difference devices (all *P* < 0.001). The 95% limits of agreement of ACD, C-ICL, vault measurements were 0.13 to 0.38 mm, − 0.07 to 0.27 mm, 0.08 to 0.34 mm between AS-OCT and Pentacam, − 0.03 to 0.33 mm, − 0.16 to 0.31 mm, − 0.10 to 0.26 mm between AS-OCT and UBM, and − 0.29 to 0.07 mm, − 0.25 to 0.20 mm, − 0.31 to 0.05 mm between Pentacam and UBM, respectively.

**Conclusions:**

AS-OCT demonstrated significantly higher value, while Pentacam demonstrated significantly lower value than UBM for ACD, C-ICL and central vault measurements in myopic eyes after ICL surgery. Measurements with these instruments were highly correlated, but could not replace each other especially for vault. This study provided valuable information about how to judge the results of anterior segment parameters of eyes implanted with ICL V4c from different devices.

**Trial registration:**

Registration number: ChiCTR-OOC-16008987. Retrospectively registered: 08 August 2016.

## Introduction

The Implantable Collamer Lens (ICL) has becoming more and more commonly used as an effective technique for correcting myopia in recent years [1, 2] . It was designed to be implanted on the ciliary sulcus in the posterior chamber, and far away from corneal endothelium. This kind of surgery may have advantages over keratorefractive surgery because it is reversible and replaceable with another ICL. Besides, ICL implantation prevents irreversible healthy corneal damage, and provides remarkably predictable and stable results with minimal surgical trauma, especially in correcting very high myopia [3].

Appropriate ICL central vault, defined as the distance between the central posterior surface of the ICL and the anterior surface of the lens capsule, is considered to be highly related to the success and safety of ICL implantation. Generally, a desirable central vault is between 0.25 mm and 0.75 mm. High vault is a potential cause of angle close and high intraocular pressure, whereas a low vault can lead to the formation of cataract [4, 5]. As a result, inappropriate ICL vault may lead to severe postoperative adverse events and is a key indication for ICL withdrawn or replacement. Besides, anterior chamber depth (ACD) is important for selecting proper ICL size and evaluating whether patients are suitable to accept ICL implantation, as ACD value less than 2.80 mm is considered inappropriate for ICL surgery. So the precise measurement of anterior segment parameters including ACD and vault is highly emphasized in ICL surgery.

Anterior segment imaging instruments have been developed to objectively visualize and evaluate anterior segment parameters. Among different devices, anterior segment optical coherence tomography (AS-OCT), Pentacam and ultrasound biomicroscopy (UBM) are most widely used. AS-OCT acquires cross-sectional images of the anterior segment without corneal contact by using low-coherence interferometry [6]. Pentacam provides section spatial planes with a rotating camera, which is modeled on the Scheimpflug principle [7]. UBM obtains objective and quantitative measurements of the anterior segment with high-definition imaging [8], although could be uncomfortable with the placement of an eyecup between the lids. Up to now, there is still no gold standard method for measuring the anterior segment parameters.

Previously, comparison of anterior segment parameters measurements among those devices have been evaluated in both health and pathology eyes. However, the results may not be suitable for evaluating the measurements after ICL implantation as the existence of ICL may interrupt the image processing system of those devices. The different refractive indices and velocities between ICL material and aqueous humor would have effect on the measurements. This study was designed to compare and correlate ACD, distance between the corneal endothelium and anterior surface of ICL(C-ICL) and central vault in myopic eyes who underwent ICL surgery for correcting myopia using three of the most advanced anterior-segment imaging systems: Visante AS-OCT, Pentacam, and high-frequency UBM. To the best of our knowledge, there has been no clinical comparison of these three methods for ACD, C-ICL and vault measurement in myopic eyes implanted with ICL V4c. This study may provide valuable information about how to judge the results of anterior segment parameters of eyes implanted with ICL V4c from different devices and help evaluate the prognosis and safety of the eyes.

## Subjects and methods

Myopic patients who had nontoric Implantable Collamer Lens (ICL, Visian V4c, STAAR Surgical, Switzerland) implantation between Jan 2016 and Aug 2016 at Eye center, Second Affiliated Hospital, School of Medicine, Zhejiang University were included in this study. This prospective study was adherent to the tenets of the Declaration of Helsinki, and was approved by the ethics committee of our hospital. All patients participating in the investigation signed the informed consent. Patients were evaluated during their regular scheduled follow up. The study has been registered in http://www.chictr.org.cn as No.ChiCTR-OOC-16008987.

### Implantable Collamer Lens power calculation and size selection

The Implantable Collamer Lens power calculations were completed by STAAR Surgical Co using a modified formula. The sizes of lens were selected depending on the corneal horizontal white-to-white value measured using corneal topography (Orbscan II; Bausch & Lomb, Rochester, NY, USA). Sulcus-to-sulcus measurements and ACD measured with UBM were also examined to reconfirm the lens size.

### Surgical technique

All the ICL implantation surgeries were performed by the same experienced surgeon (YB.Y.) according to the standard procedures. In brief, the patients were given dilating and cycloplegic agents on the day of surgery. Topical anesthesia was done with the administration of one drop of 0.5% Proparacaine Hydrochloride Eye Drops (Alcaine, Alcon, USA) 3 times every 5 min before surgery. After injecting viscoelastic agent into the anterior chamber to maintain the operating space, a model V4c Implantable Collamer Lens loaded in an injector cartridge (Staar Surgical, Switzerland) was inserted into the anterior chamber through a 3.0 mm clear corneal incision. The ICL was then placed in the posterior chamber, and the remaining viscoelastic agent in front of and behind ICL was completely washed out with balanced salt solution. All the surgeries were performed without complications. After surgery, 0.5% levofloxacin eye drops (Cravit, Santen Pharmaceutical, Japan) and 0.1% fluorometholone eye drops (Flumetholon, Santen Pharmaceutical, Japan) were topically administered four times daily for 2 weeks.

### Anterior segment parameters measurement

**Fig. 1:**
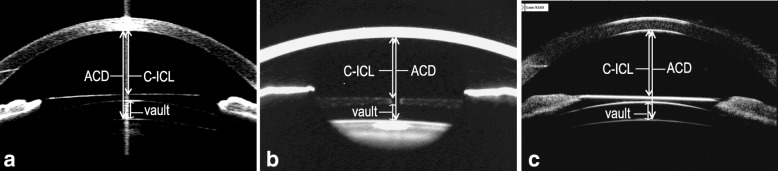
Anterior segment measured by AS-OCT (**a**), Pentacam (**b**) and UBM (**c**) in phakic eyes after ICL implantation. ACD: Anterior chamber depth; C-ICL: Distance between the corneal endothelium and anterior surface of ICL; Vault: Distance between the back surface of ICL and the front surface of the lens

Postoperative anterior segment parameters (including ACD, C-ICL and vault) were measured using AS-OCT, Pentacam and UBM in sequence the day at 3 months follow up. A single experienced operator performed all the examinations, which were then assessed and analyzed by other researchers. The horizontal median images were captured for analysis, because eyelid may cover the superior and inferior limbuses as well as distort the anterior segment structure. The measurements were obtained using the on-screen calibration system. The ACD was measured from the center of the corneal endothelial to the anterior surface of the lens capsule. C-ICL was measured from the central corneal endothelium and anterior surface of ICL. The vault was defined as the central distance from the back surface of the ICL to the anterior surface of the lens capsule (Fig. 1). Two independent measurements were taken at the respective instrument to assess the repeatability of the instruments. The first measurement was used for the comparative analysis.

AS-OCT (Model 1000, Carl Zeiss Meditec, USA) was conducted in dark environment with the patients seated and eyes fogged with internal lens to avoid accommodation. It captured and provided cross-sectional images of the anterior segment with an axial resolution of 18 μm and transversal resolution of 60 μm. In order to prevent image deformity, the eye was guided by an internal flashing light to keep the limbal surface perpendicular to the coherence light beam during examination.

Pentacam (Oculus, Germany) was also performed in a dark room with the patients seated and the eye guided by an internal target for fixation. During the measurement, a camera rotated and acquired 3-dimensional scan of the anterior eye segment to recreate a 3D image with maximum 138,000 evaluated measuring points, from which the horizontal median parameters were measured.

UBM (SW-3200, Suoer electric Industries, China) examination, using a 50-MHz transducer probe with pure water as the coupling agent, was performed with the patients in a supine position. Both axial and transversal resolution of UBM is 50 μm. Topical anesthesia was necessary because an eye cup of suitable size was required to be put into the conjunctival sac during the examination. The effect of accommodation was tried to be prevented by the contralateral eye fixing on a target on the ceiling. Images of horizontal anterior segment were acquired by keeping the probe perpendicular to the horizontal limbus, with care to avoid pressing the globe or touching the cornea.

## Statistical analysis

All data sets were normally distributed according to the results of Kolmogorov-Smirnov test (*p*>0.05) and were presented as mean ± standard deviation (SD). The repeatability of instruments was analyzed by Pearson correlation and Paired-t test. The differences between the instruments in ACD, C-ICL and vault measurements were examined by Paired–t tests as follows: AS-OCT vs Pentacam, AS-OCT vs UBM, and Pentacam vs UBM. The correlation of parameters measured with different instruments was evaluated by Pearson correlation coefficient (r). The reliability of measurements from different instruments was calculated by intraclass correlation coefficient (ICC). In addition, the agreement of the measurement procedures was assessed by Bland and Altman plot. The variability of the measured values were described as the 95% limits of agreement (95%LOA = mean ± 1.96 SD). All tests were two tailed analyzed, with *P* values less than 0.05 considered statistically significant.

## Results

This study included 82 eyes of 82 patients (31 males, 51 females) underwent ICL implantation. The mean age was 27.1 ± 4.7 years (range, 21 to 42 years). The pre-operative spherical equivalent (SE) was − 10.1 ± 4.0 D (range, − 2.25 to − 22.5D), implanted ICL power was − 11.4 ± 3.6D (range, − 3 to -18D), preoperative intraocular pressure (IOP) was 15.3 ± 2.3 mmHg (range, 10.5 to 20.7 mmHg), and postoperative IOP was 14.3 ± 2.7 mmHg (range, 8.3 to 19.3 mmHg). There was no complication occurred or observed during the surgery and follow up time.

Two independent measurements were taken at the respective instrument to assess the repeatability of the instruments. Table 1 showed that all of Pentacam, UBM and AS-OCT had high repeatability and accuracy in measuring ACD, C-ICL and vault.

**Table 1:** Repeatability of Pentacam, UBM and AS-OCT in measuring ACD, C-ICL and Vault

Device	Parameter	Pearson (r)	*P*-value	Difference(mm)	t-value	*P*-value
AS-OCT	ACD	0.98	0.000	0 ± 0.04	−1.20	0.23
	C-ICL	0.99	0.000	0 ± 0.04	−0.48	0.64
	Vault	0.99	0.000	0 ± 0.03	1.44	0.15
Pentacam	ACD	0.98	0.000	0 ± 0.04	−0.85	0.40
	C-ICL	0.99	0.000	0. ± 0.04	−0.90	0.37
	Vault	0.99	0.000	0 ± 0.03	−0.77	0.45
UBM	ACD	0.98	0.000	0 ± 0.05	0.36	0.72
	C-ICL	0.99	0.000	0 ± 0.05	0.83	0.41
	Vault	0.99	0.000	0 ± 0.04	0.22	0.83

AS-OCT: Anterior segment optical coherence tomography; UBM: Ultrasound biomicroscopy; Pearson (r): Pearson correlation coefficient; ACD: Anterior chamber depth; C-ICL: Distance between the corneal endothelium and anterior surface of ICL; Vault: Distance between the back surface of ICL and the front surface of the lens. *P* values less than 0.05 were considered statistically significant

### Comparison of measurements between instruments

The mean values and comparison of anterior section parameters including ACD, C-ICL and vault from 3 instruments were summarized in Table 2 and Table 3. As compared with both of Pentacam and UBM, AS-OCT showed statistically significant higher values for all measurements (*P* < 0.001 for all). At the same time, Pentacam provided statistically smaller values for all measurements than UBM (*P* < 0.001, *P* = 0.031, and *P* < 0.001 for ACD, C-ICL and vault respectively).

**Table 2:** Measurements of anterior section parameters using AS-OCT, Pentacam and UBM

Parameters(Mean ± SD)	AS-OCT(mm)	Pentacam(mm)	UBM(mm)
ACD	3.18 ± 0.23	2.93 ± 0.22	3.04 ± 0.22
C-ICL	2.32 ± 0.28	2.22 ± 0.27	2.24 ± 0.29
Vault	0.64 ± 0.25	0.43 ± 0.24	0.56 ± 0.27

**Table 3:** Comparison of anterior section parameters measurements using AS-OCT, Pentacam and UBM

Parameter	AS-OCT vs Pentacam			AS-OCT vs UBM			Pentacam vs UBM		
	ACD	C-ICL	Vault	ACD	C-ICL	Vault	ACD	C-ICL	Vault
t	35.3	10.64	27.67	14.37	5.47	7.81	−10.87	−2.19	−12.67
*P*	0.000	0.000	0.000	0.000	0.000	0.000	0.000	0.031	0.000

Paired t-tests were used to compare measurements. *P* values less than 0.05 were considered statistically significant

### Reliability and agreement between instruments

The ICC results in Table 4 indicated high reliability of 3 instruments for anterior section parameters measurements (all *P*<0.001). The Pearson correlation coefficient in Table 4 also showed that statistically significant correlation were found between different instruments for measuring ACD, C-ICL and central vault (all *P*<0.001).

**Table 4:** Results of ICC and Pearson correlation coefficient (r) for each parameter between different devices

Parameter	AS-OCT vs Pentacam			AS-OCT vs UBM			Pentacam vs UBM		
	ACD	C-ICL	Vault	ACD	C-ICL	Vault	ACD	C-ICL	Vault
ICC	0.98	0.98	0.98	0.96	0.95	0.97	0.96	0.96	0.97
*P*(ICC)	0.000	0.000	0.000	0.000	0.000	0.000	0.000	0.000	0.000
Pearson (r)	0.96	0.95	0.96	0.92	0.91	0.94	0.91	0.92	0.94
*P* (r)	0.000	0.000	0.000	0.000	0.000	0.000	0.000	0.000	0.000

ICC: Intraclass correlation coefficient. *P* values less than 0.05 were considered statistically significant

**Fig. 2:**
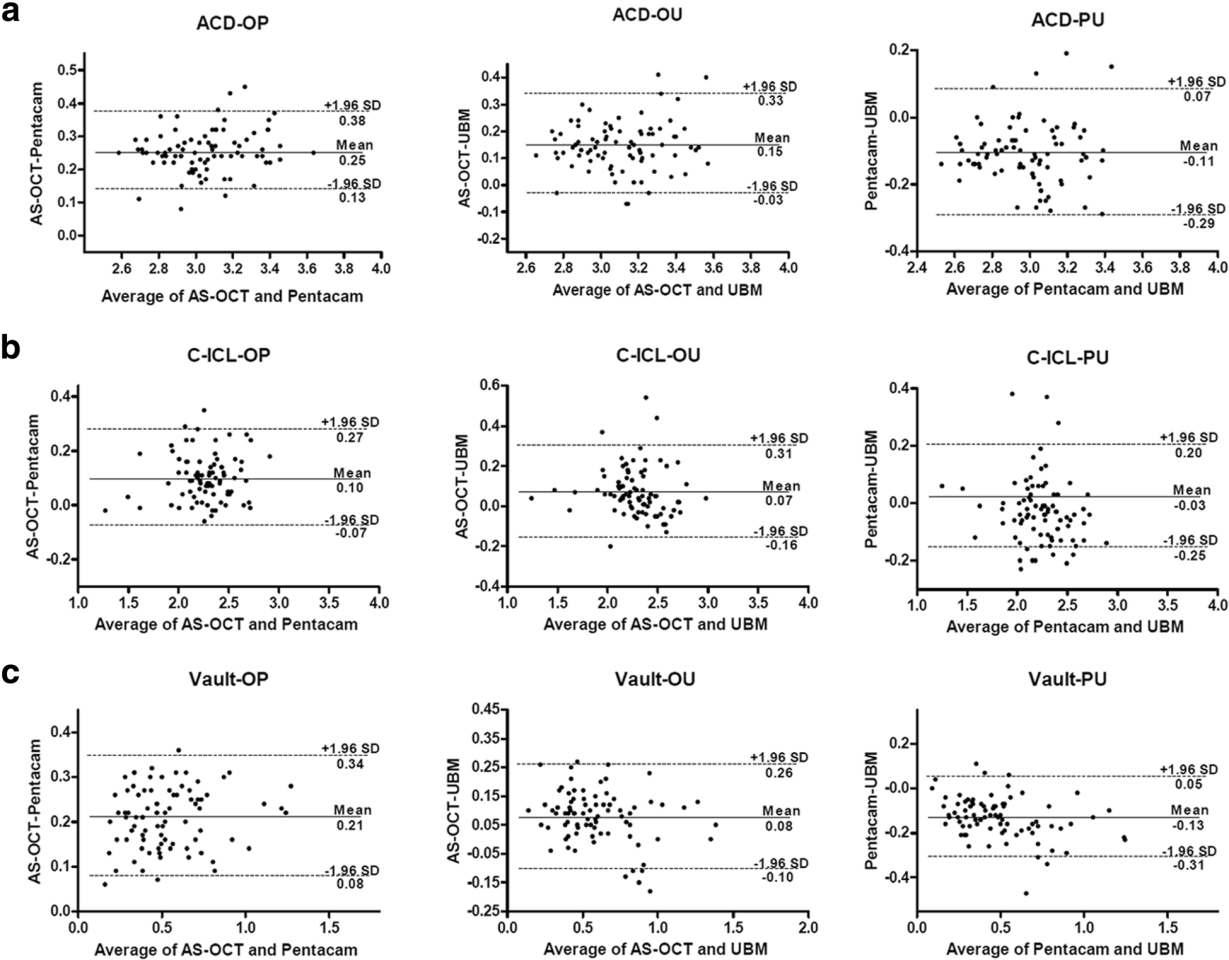
Bland and Altman plots comparing the level of agreement between the 3 instruments for ACD (**a**), C-ICL (**b**), Vault (**c**) measurements. The vertical axis represents the difference between these measurements and the horizontal axis shows their mean. Dashed lines represent the 95% confidence intervals. Solid line represents the mean difference. All scales in mm. ACD: Anterior chamber depth; C-ICL: Distance between the corneal endothelium and anterior surface of ICL; Vault: Distance between the back surface of ICL and the front surface of the lens

Agreement of ACD, C-ICL and vault measurements between different methods were illustrated using Bland-Altman plots (Fig. 2). The mean differences between measurements from AS-OCT, Pentacam and UBM together with 95% limits of agreement were shown in Table 5.

**Table 5:** Agreement of parameters measured by AS-OCT, Pentacam and UBM

Parameter(mm)	AS-OCT vs Pentacam Mean (95%)	AS-OCT vs UBM Mean (95%)	Pentacam vs UBM Mean (95%)
ACD	0.25 (0.13, 0.38)	0.15 (−0.03, 0.33)	−0.11 (− 0.29, 0.07)
C-ICL	0.10 (− 0.07, 0.27)	0.07 (− 0.16, 0.31)	−0.03 (− 0.25, 0.20)
vault	0.21 (0.08, 0.34)	0.08 (− 0.10, 0.26)	−0.13 (− 0.31, 0.05)

Mean (95%): mean value of measurement difference between 2 methods (95% limits of agreement)

## Discussion

In this study, we examined the repeatability of different devices as it is an important factor when selecting an instrument for clinical purposes. It showed that all of AS-OCT, Pentacam and UBM had good repeatability for measuring ACD, C-ICL and vault. Then, we compared and correlated ACD, C-ICL and vault measurements between AS-OCT, Pentacam and UBM in high myopia eyes after ICL implantation. Not surprisingly, our results were not highly consistent with others’, as the results of several previous studies comparing anterior segment parameter measurements among different devices have also shown contrary results.

Several [9–11] studies found no statistically significant difference between AS-OCT and Pentacam for ACD measurement. On contrary, Wang [12], Fu. [13], O’Donnell [14] and Nemeth [15] observed that Visante AS-OCT measured significantly deeper ACD values by a mean of 0.07 to 0.2 mm than Pentacam, but the first two with good and the other two with poor agreement between AS-OCT and Pentacam. Similarly, Nuijts [16] found that AS-OCT provided higher ACD measurements than Pentacam in both of healthy people and pIOL patients. Although contradictions existed, limits of agreement results obtained in those studies showed that AS-OCT and Pentacam could be used interchangeably for calculating IOL power but not for estimating IOL vault. In our study, ACD values measured with AS-OCT and Pentacam correlated well, but AS-OCT measurements were significantly higher as compared with Pentacam by a mean of 0.25 mm. The 95% LoA between the two instruments was 0.13 to 0.38 mm, which was not clinically acceptable. Our results indicated that AS-OCT and Pentacam could not be used interchangeably for ACD measurement after ICL surgery.

Many researchers have assessed the difference of ACD measurements between AS-OCT and UBM. Dada [17], Pinero [18] and Zhang [19] found that AS-OCT measured a little bit higher ACD values by a mean of 0.07–0.09 mm than UBM in phakic eyes without statistically significant differences, and the measurements agreement were well [18, 19]. However, AS-OCT measured significantly deeper ACD than UBM in phakic eyes as reported by Nemeth [20]. Similaily, Zhang [19] found that the ACD measurement with AS-OCT was significantly higher compared with UBM in pseudophakic eyes after cataract surgery, and the 95% LoA of ACD measurements was − 0.32 to 0.62 mm. The authors [19] concluded that although the ICC was high, AS-OCT and UBM could not be used interchangeably for ACD measurements in pseudophakic eyes. Furthermore, Zhang [21] compared ACD measurement with AS-OCT and UBM in eyes after ICL V4 implantation. The results showed that AS-OCT slightly but significantly overestimated ACD by a mean of 0.058 mm than UBM. Consistent with Zhang’s results, our findings showed that the ACD measured by AS-OCT was significantly higher by a mean of 0.15 mm than that measured by UBM. Good correlation was found between these two devices, but the 95% LOA between two devices was − 0.03 to 0.33 mm, which was also not clinically acceptable.

Up to now, much less research has compared the ACD measurement between UBM and Pentacam, and their results also presented different findings. Nakakura [22] found that Pentacam measured significantly higher ACD value than UBM in healthy people. Yu [23] reported no significant difference between Pentacam and UBM in highly myopic eyes. On contrary, Nemeth [20] showed that Pentacam and UBM measured the same ACD values in phakic eyes, but Pentacam measured significantly shallower ACD values in pseudophakic eyes. Similar to Nemeth’s results, we found that ACD measured by UBM was significantly higher than that measured by Pentacam. UBM and Pentacam showed statistically significant good correlation, but the 95% LOA was − 0.29 to 0.07 mm which was also clinically unacceptable.

As referred to C-ICL and vault measurements comparation among AS-OCT, Pentacam and UBM, there has been limited studies so far. Zhangs’ [21] reported that AS-OCT gave a comparable C-ICL measurements but significantly higher vault measurement than UBM in eyes after ICL implantation. A significantly good correlation was found between OCT and UBM, but the 95% LoA of vault measurements indicated that the difference between these two devices was clinically unacceptable. Wang and Lu reported that vault measurements of Pentacam are smaller than UBM [24, 25], but without statistical significance. The limited case numbers in those previous studies may make it difficult to find the subtle difference between different methods. In our study, AS-OCT measured significantly deeper C-ICL and vault values by a mean of 0.07 and 0.08 mm than UBM respectively with good measurements correlation. The 95% LoA of C-ICL and vault measurements between these two instruments were − 0.16 to 0.31 mm and − 0.10 to 0.26 mm, respectively. Additionally, we found that both of AS-OCT and UBM measured significantly deeper C-ICL and central vault values than Pentacam, with good measurements correlation. The 95% LoA of C-ICL, vault measurements were − 0.07 to 0.27 mm and 0.08 to 0.34 mm between AS-OCT and Pentacam, and − 0.25 to 0.20 mm and − 0.31 to 0.05 mm between Pentacam and UBM, respectively. As a desirable vault height is between 0.25 and 0.75 mm [26], our study indicated that AS-OCT, UBM and Pentacam could not be used interchangeably for postoperatively evaluating the safety of high myopia patient after ICL implantation, especially for measuring vault.

In our study, there is a tendency that AS-OCT had slightly larger, while Pentacam had smaller values than UBM. Several reasons might explain the discrepancy among three instruments. Firstly, AS- OCT, Pentacam and UBM each have different resolution. It is possible that the difference in resolution among instruments leads to the measurement difference. Secondly, the supine positioning and the potential pressure from the eye cup and probe might distort the original configuration of the anterior segment during UBM examination [27]. Thirdly, the difference of scan location may contribute to the disagreement among instruments. OCT and Pentacam are designed to be able to precisely control the scan direction because the cornea was divided to 360 degrees. On contrary, the scan direction and reproducibility of UBM depend on the operator’s experience and patient’s cooperation. Moreover, different from AS-OCT and Pentacam, the UBM probe tip is too large to locate the exact target limbal site. Fourthly, the disagreements may result from the different image processing program of the 3 devices. Unlike UBM depends on mechanical pulses of sound, AS-OCT uses low coherence interferometry to evaluate the structure of eye anterior segment. On the other hand, Pentacam is modeled on the Scheimpflug principle which takes images to recreate a 3D model. Thus, the different image capture mechanism at different devices may distort the actual structure. Besides, existence of ICL may interrupt the image processing system of those devices. The different refractive indices between ICL material and aqueous humor would have effect on the measurement of all structures lying behind the ICL with AS-OCT and Pentacam, while UBM measurement will also be affected by the difference of velocities between ICL material and aqueous humor. So far, there is no specific modulation procedures designed for anterior segment parameter measurement after ICL implantation in all of these three devices. Finally, the different sources of guidance light may contribute to the discrepancy among devices. UBM requires patients to fix the external target, while Pentacam and AS-OCT use inward aiming light of different sources. The aiming light of Pentacam is brighter and keeps flickering, which may induce pupil contraction and accommodation. On the other hand, AS-OCT examination is guided by a more gentle yellow light, which do not significantly stimulate patient’s accommodation. As a result, all these factors may cause the discrepancy of measurement among AS-OCT, Pentacam and UBM.

As we all know, UBM has been widely used for the past 20 years as it could precisely show anterior segment structure with high resolution. In our study, UBM provided high quality pictures which clearly showed the contour of ICL. However, UBM is unsuitable to be used shortly after ICL surgery as it is an invasive examination. AS-OCT and Pentacam, both as non contact imaging devices, are more comfortable for patient, especially shortly after surgery. Based on our results, all of AS-OCT, Pentacam and UBM could provide objective ways for anterior segment parameters measurements after ICL implantation. Although we could not determine which one was more accurate as lacking of a gold standard method, we have shown how to judge the results from different devices. Besides, as the existence of ICL may interrupt the image processing system of those devices, further researches are needed to figure out how to eliminate the effect of ICL on measurement.

## Conclusions

In this study, AS-OCT demonstrated a significantly higher value, while Pentacam demonstrated a significantly lower value than UBM for ACD, C-ICL and vault measurements in myopic eyes after ICL surgery. Anterior segment measurement with these three instruments were highly correlated, but could not be used interchangeably especially for measuring vault. Herein we presented the results of anterior segment parameters measurements comparison among distinct techniques with the existance of ICL, which may interrupt the image processing system as a result of the different refractive indices and velocities between ICL material and aqueous humor. As different devices may be used in various eye centers, it would help judge the results of anterior segment parameters of eyes with ICL V4c implantation from different devices and would help evaluate the prognosis and safety of the eyes.

## Data Availability

The datasets used and/or analysed during the current study are available from the corresponding author on reasonable request.

## Abbreviations

95%LOA: 95% limits of agreement
ACD: Anterior chamber depth
AS-OCT: Anterior segment optical coherence tomography
C-ICL: Distance between the corneal endothelium and anterior surface of ICL
ICC: Intraclass correlation coefficient
ICL: Implantable Collamer lens
Mean (95%): mean value of measurement difference between 2 methods (95% limits of agreement)
Pearson (r): Pearson correlation coefficient
SD: Standard deviation
UBM: Ultrasound biomicroscopy

## References

[1] Fernandez-Vega-Cueto L, Lisa C, Esteve-Taboada JJ, Montes-Mico R, Alfonso JF (2018). Implantable collamer lens with central hole: 3-year follow-up. Clin Ophthalmol.

[2] Miao H, Chen X, Tian M, Chen Y, Wang X, Zhou X (2018). Refractive outcomes and optical quality after implantation of posterior chamber phakic implantable collamer lens with a central hole (ICL V4c). BMC Ophthalmol.

[3] Kamiya K, Shimizu K, Igarashi A, Hikita F, Komatsu M (2009). Four-year follow-up of posterior chamber phakic intraocular lens implantation for moderate to high myopia. Arch Ophthalmol.

[4] Chung TY, Park SC, Lee MO, Ahn K, Chung ES (2009). Changes in iridocorneal angle structure and trabecular pigmentation with STAAR implantable collamer lens during 2 years. J Refract Surg.

[5] Alfonso JF, Lisa C, Fernandez-Vega L, Almanzar D, Perez-Vives C, Montes-Mico R (2015). Prevalence of cataract after collagen copolymer phakic intraocular lens implantation for myopia, hyperopia, and astigmatism. J Cataract Refract Surg.

[6] Jiao H, Hill LJ, Downie LE, Chinnery HR. Anterior segment optical coherence tomography: its application in clinical practice and experimental models of disease. Clin Exp Optom. 2018. 10.1111/cxo.12835 30270476

[7] Guo JM, Li M, Xu XL, Zhang H, Wang JM (2015). Anterior segment changes after pharmacologic mydriasis using Pentacam and optical coherence tomography in angle closure suspects. Int J Ophthalmol..

[8] Unsal E, Eltutar K, Muftuoglu IK (2017). Morphologic changes in the anterior segment using ultrasound biomicroscopy after cataract surgery and intraocular lens implantation. Eur J Ophthalmol.

[9] Shajari M, Lehmann UC, Kohnen T (2016). Comparison of corneal diameter and anterior chamber depth measurements using 4 different devices. Cornea..

[10] Dinc UA, Gorgun E, Oncel B, Yenerel MN, Alimgil L (2010). Assessment of anterior chamber depth using Visante optical coherence tomography, slitlamp optical coherence tomography, IOL master, Pentacam and Orbscan IIz. Ophthalmologica..

[11] Yazici AT, Bozkurt E, Alagoz C, Alagoz N, Pekel G, Kaya V (2010). Central corneal thickness, anterior chamber depth, and pupil diameter measurements using Visante OCT, Orbscan, and Pentacam. J Refract Surg.

[12] Wang Q, Ding X, Savini G, Chen H, Feng Y, Pan C (2015). Anterior chamber depth measurements using Scheimpflug imaging and optical coherence tomography: repeatability, reproducibility, and agreement. J Cataract Refract Surg.

[13] Fu J, Wang X, Li S, Wu G, Wang N (2010). Comparative study of anterior segment measurement with Pentacam and anterior segment optical coherence tomography. Can J Ophthalmol.

[14] O'Donnell C, Hartwig A, Radhakrishnan H (2012). Comparison of central corneal thickness and anterior chamber depth measured using LenStar LS900, Pentacam, and Visante AS-OCT. Cornea..

[15] Nemeth Gabor, Hassan Ziad, Szalai Eszter, Berta Andras, Modis Laszlo (2013). Anterior Segment Parameters Measured with 2 Optical Devices Compared to Ultrasonic Data. European Journal of Ophthalmology.

[16] Doors M, Cruysberg LP, Berendschot TT, de Brabander J, Verbakel F, Webers CA (2009). Comparison of central corneal thickness and anterior chamber depth measurements using three imaging technologies in normal eyes and after phakic intraocular lens implantation. Graefes Arch Clin Exp Ophthalmol.

[17] Dada T, Sihota R, Gadia R, Aggarwal A, Mandal S, Gupta V (2007). Comparison of anterior segment optical coherence tomography and ultrasound biomicroscopy for assessment of the anterior segment. J Cataract Refract Surg.

[18] Pinero DP, Plaza AB, Alio JL (2008). Anterior segment biometry with 2 imaging technologies: very-high-frequency ultrasound scanning versus optical coherence tomography. J Cataract Refract Surg.

[19] Zhang Q, Jin W, Wang Q (2010). Repeatability, reproducibility, and agreement of central anterior chamber depth measurements in pseudophakic and phakic eyes: optical coherence tomography versus ultrasound biomicroscopy. J Cataract Refract Surg.

[20] Nemeth G, Tsorbatzoglou A, Modis L, Berta A (2009). Orv Hetil.

[21] Zhang J, Luo HH, Zhuang J, Yu KM (2016). Comparison of anterior section parameters using anterior segment optical coherence tomography and ultrasound biomicroscopy in myopic patients after ICL implantation. Int J Ophthalmol.

[22] Nakakura S, Mori E, Nagatomi N, Tabuchi H, Kiuchi Y (2012). Comparison of anterior chamber depth measurements by 3-dimensional optical coherence tomography, partial coherence interferometry biometry, Scheimpflug rotating camera imaging, and ultrasound biomicroscopy. J Cataract Refract Surg.

[23] Yu AY, Lin ZD, Chen XQ, Cai XY, Liu YZ, Luo SK (2008). Position of myopic iris-claw phakic intraocular lens by Scheimpflug photography and ultrasound biomicroscopy. Eye (Lond).

[24] Zhang Xi WX (2015). Comparative analysis of preoperative simulated vaults and postoperative real vaults in implantable collamer lens implantation. Chin J Optom Ophthalmol Vis Sci.

[25] Lu Y. Five year follow up of the changes in central vaulting and thickness of the crystalline lens after phakic collamer lens(ICL) implantation: China Medical University; 2014. p. 38.

[26] Kamiya K, Shimizu K, Komatsu M (2009). Factors affecting vaulting after implantable collamer lens implantation. J Refract Surg.

[27] Ishikawa H, Inazumi K, Liebmann JM, Ritch R (2000). Inadvertent corneal indentation can cause artifactitious widening of the iridocorneal angle on ultrasound biomicroscopy. Ophthalmic Surg Lasers.

